# Improving the process of research ethics review

**DOI:** 10.1186/s41073-017-0038-7

**Published:** 2017-08-18

**Authors:** Stacey A. Page, Jeffrey Nyeboer

**Affiliations:** 1 Department of Community Health Sciences, Cumming School of Medicine, University of Calgary, Calgary, Alberta Canada; 2 Conjoint Health Research Board, University of Calgary, Calgary, Alberta Canada; 3 ITM Vocational University, Vadodara, Gujurat India

**Keywords:** Research ethics, Research Ethics Boards, Research Ethics Committees, Medical research, Applied ethics, Institutional Review Boards

## Abstract

**Background:**

Research Ethics Boards, or Institutional Review Boards, protect the safety and welfare of human research participants. These bodies are responsible for providing an independent evaluation of proposed research studies, ultimately ensuring that the research does not proceed unless standards and regulations are met.

**Main body:**

Concurrent with the growing volume of human participant research, the workload and responsibilities of Research Ethics Boards (REBs) have continued to increase. Dissatisfaction with the review process, particularly the time interval from submission to decision, is common within the research community, but there has been little systematic effort to examine REB processes that may contribute to inefficiencies. We offer a model illustrating REB workflow, stakeholders, and accountabilities.

**Conclusion:**

Better understanding of the components of the research ethics review will allow performance targets to be set, problems identified, and solutions developed, ultimately improving the process.

## Background

Instances of research misconduct and abuse of research participants have established the need for research ethics oversight to protect the rights and welfare of study participants and the integrity of the research enterprise [1, 2]. In response to such egregious events, national and international regulations have emerged that are intended to protect research participants (e.g. [3–5]).

Research Ethics Boards (REBs) also known as Institutional Review Boards (IRBs) and Research Ethics Committees (RECs) are charged with ensuring that research is planned and conducted in accordance with such laws and regulatory standards. In protecting the rights and welfare of participants, REBs must weigh possible harms to individuals against the plausible societal benefits of the research. They must ensure fair participant selection and, where applicable, confirm that appropriate provisions are in place for obtaining participant consent.

REBs often operate under the auspices of post-secondary institutions. Larger universities may support multiple REBs that serve different research areas, such as medical and health research and social science, psychology, and humanities research. Boards are constituted of people from a variety of backgrounds, each of whom contributes specific expertise to review and discussions. Members are appointed to the Board through established institutional practice. Nevertheless, most Board members bring a sincere interest and commitment to their roles. For university Faculty, Board membership may fulfil a service requirement that is part of their academic responsibilities.

The Canadian Tri-Council Policy Statement (TCPS2) advances a voluntary, self-governing model for REBs and institutions. The TCPS2 is a joint policy of Canada’s three federal research agencies (Canadian Institutes of Health Research, Natural Sciences and Engineering Research Council of Canada, and Social Sciences and Humanities Research Council), and institutional and researcher adherence to the policy standards is a condition of funding. Recognizing the independence of REBs in their decision-making, institutions are required to support their functioning. Central to the agreement is that institutions conducting research must establish an REB and ensure that it has the “necessary and sufficient ongoing financial and administrative resources” to fulfil its duties (TCPS2 [3] p. 68). A similar requirement for support of IRB functioning is included in the US Common Rule (45 CFR 46.103 [5]). The operationalization of “necessary and sufficient” is subjective and likely to vary widely. To the extent that the desired outcomes (i.e. timely reviews and approvals) depend on the allocation of these resources, they too will vary.

## Time and research ethics review

From the academic hallways to the literature, characterizations of REBs and the research ethics review process are seldom complimentary. While numerous criticisms have been levelled, it is the time to decision that is most consistently maligned [6–11].

Factors associated with lengthy review time include incomplete or poorly completed applications [7, 12, 13], lack of administrative support [14], inadequately trained REB members [15], REB member competing commitments, expanding oversight requirements, and the sheer volume of applications [16–18]. Nevertheless, objective data on the inner workings of REBs are lacking [6, 19, 20].

Consequences of slow review times include centres’ withdrawing from multisite trials or limiting their participation in available trials [21, 22], loss of needed research resources [23], and recruitment challenges in studies dependent on seasonal factors [24]. Lengthy time to study approval may ultimately delay patient access to potentially effective therapies [8].

Some jurisdictions have moved to regionalize or consolidate ethics review, using a centralized ethics review of protocols conducted on several sites. This enhances review efficiency for multisite research by removing the need for repeating reviews across centres [9, 25–28]. Recommendations for systemic improvement include better standardization of review practices, enhanced training for REB members, and requiring accreditation of review boards [9].

The research ethics review processes are not well understood, and no gold standard exists against which to evaluate board practices [19, 20]. Consequently, there is little information on how REBs may systematically improve their methods and outcomes. This paper presents a model based on stakeholder responsibilities in the process of research ethics review and illustrates how each makes contributions to the time an application spends in this process. This model focusses on REBs operating under the auspices of academic institutions, typical in Canada and the USA.

## Modelling the research ethics review process

The research ethics review process may appear to some like the proverbial black box. An application is submitted and considered and a decision is made:

SUBMIT > REVIEW > DECISION

In reality, the first step to understanding and improving the process is recognizing that research ethics review involves more than just the REB. Contributing to the overall efficiency—or inefficiency—of the review are other stakeholders and their roles in the development and submission of the application and the subsequent movement of the application back and forth between PIs, administrative staff, reviewers, the Board, and the Chair, until ideally the application is deemed ready for approval.

Identifying how a research ethics review progresses permits better understanding of the workflow, including the administrative and technological supports, roles, and responsibilities. The goal is to determine where challenges in the system exist so they can be remediated and efficiencies gained.

One way of understanding details of the process is to model it. We have used a modelling approach based in part on a method advanced by Ishikawa and further developed by the second author (JN) [29, 30]. Traditionally, the Ishikawa “fishbone” or cause and effect diagram has been used to represent the components of a manufacturing enterprise and its application facilitates understanding how the elements of an operation may cause inefficiencies. This modelling provides a means of analysing process dispersion (e.g. who is accountable for what specific outcomes) and is frequently used when trying to understand time delays in undertakings.

**Fig. 1:**
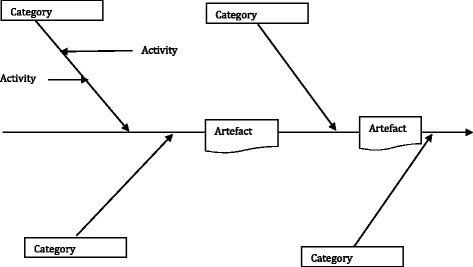
Basic business activity model

In our model (Fig. 1), “Categories” represent key role actions that trigger a subsequent series of work activities. The “Artefacts” are the products resulting from a set of completed activities and reflect staged movement in the process. Implicit in the model is a temporal sequence and the passage of time, represented by the arrows.

Applying this strategy to facilitate understanding of time delays in ethics review requires that the problem (i.e. time) be considered in the context of all stakeholders. This includes those involved in the development and submission of the application, those involved in the administrative movement of the application through the system, those involved in the substantive consideration and deliberation of the application, and those involved in the final decision-making.

**Fig. 2:**
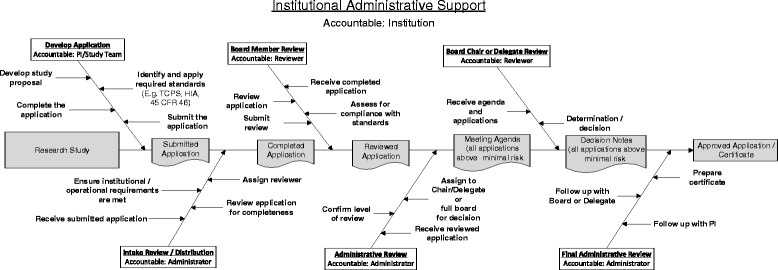
Research ethics activity model

The model developed (Fig. 2) was based primarily on a review of the lead author’s (SP) institution’s REB application process. The model is generally consistent with the process and practices of several other REBs with which she has had experience over the past 20 years.

What this model illustrates is that the research ethics review process is complex. There are numerous stakeholders involved, each of whom bears a portion of the responsibility for an application’s time in the system. The model illustrates a temporal sequence of events where, ideally, the movement of an application is unidirectional, left to right. Time is lost when applications stall or backflow in the process.

## Stakeholders, accountabilities, and the research ethics review model

There are four main stakeholder groups in the research ethics review process: researchers/research teams, research ethics unit administrative staff, REB members, and the institution. Each plays a role in the transit of an application through the process and how well they undertake their role responsibilities affects the time that the application takes to move through. Table 1 presents a summary of recommendations for best practices.

**Table 1:** Best practice recommendations

Stakeholder	Activity
Researchers	• Develop scientifically sound research proposals • Understand and apply research ethics standards • Ensure that applications are thorough and complete • Be responsive to requests for revision and clarification
Research administrators	• Understand and apply institutional requirements • Understand and be able to communicate research ethics standards • Share observed challenges with the appropriate institutional delegate so that solutions can be developed
Research Ethics Board members	• Understand and consistently apply research ethics standards • Respect review timelines • Participate in (ongoing) educational activities
Institution	• Provide necessary administrative support, responsive to variation in workload • Encourage and support educational opportunities for researchers, administrators and REB members • Promote a culture of respect for research ethics review

### Researchers

The researcher initiates the process of research ethics review by developing a proposal involving human participants and submitting an application. Across standards, the principal investigator is accountable for the conduct of the study, including adherence to research ethics requirements. Such standards are readily available both from the source (e.g. Panel on Research Ethics [Canada], National Institutes of Health [USA], Food and Drug Administration [USA]) and, typically, through institutional websites. Researchers have an obligation to be familiar with the rules for human participant research. Developing a sound proposal where ethics requirements are met at the outset places the application in a good position at the time of submission. Researchers are accountable for delays in review when ethical standards are not met and the application must be returned for revision. Tracking the reasons for return permits solutions, such as targeted educational activities, to be developed.

Core issues that investigators can address in the development of their applications include an ethical recruitment strategy, a sound consent process, and application of relevant privacy standards and legislation. Most research ethics units associated with institutions maintain websites where key information and resources may be found, such as consent templates, privacy standards, “frequently asked questions,” and application submission checklists [31–33]. Moreover, consulting with the REB in advance of submission may help researchers to prevent potentially challenging issues [15]. Investigators who are diligent in knowing about and applying required standards will experience fewer requests for revision and fewer stalls or backtracking once their applications are submitted. Some have suggested that researchers should be required, rather than merely expected, to have an understanding of legal and ethics standards before they are even permitted to submit an application [19].

The scholarly integrity of proposed research is an essential element of ethically acceptable human participant research. Researchers must be knowledgeable about the relevant scientific literature and present proposals that are justified based on what is known and where knowledge gaps exist. Research methods must be appropriate to the question and studies adequately powered. Novice or inexperienced researchers whose protocols have not undergone formal peer review (e.g. via supervisory committees, internal peer review committees, or competitive grant reviews) should seek consultation and informal peer review prior to ethics review to ensure the scientific validity of their proposals. While it is within the purview of REBs to question methods and design, it is not their primary mandate. Using REB resources for science review is an opportunity cost that can compromise efficient ethics review.

Finally, researchers are advised to review and proof their applications prior to submission to ensure that all required components have been addressed and the information in the application and supporting documents (e.g. consent forms, protocol) is consistent. Missing or discrepant information is causal to application return and therefore to time lost [7].

### Administrators

Prior to submission, administrators may be the first point of contact for researchers seeking assistance with application requirements. Subsequently, they are often responsible for undertaking a preliminary, screening review of applications to make sure they are complete, with all required supporting documents and approvals in place. Once an application is complete, the administrative staff assign it to a reviewer. The reviewer may be a Board member or a subject-matter expert accountable to the Board.

Initial consultation and screening activities work best when staff have good knowledge of both institutional application requirements and ethics standards. Administrative checklists are useful tools to help ensure consistent application of standards in this preliminary application review. Poorly screened applications that reach reviewers may be delayed if the application must be returned to the administrator or the researcher for repair.

Reviewers typically send their completed reviews back to the administrators. In turn, the administrators either forward the applications to the Chair to consider (i.e. for delegated approval) or to a Board meeting agenda. In addition to ensuring that applications are complete, administrators may be accountable for monitoring how long a file is out for review. When reviews are delayed or incomplete for any reason, administrators may need to reassign the file to a different reviewer.

Administrators are therefore key players in the ethics review process, as they may be both initial resources for researchers and subsequently facilitate communication between researchers and Board members. Moreover, given past experience with both research teams and reviewers, they may be aware of areas where applicants struggle and when applications or reviews are likely to be deficient or delinquent. Actively tracking such patterns in the review process may reveal problems to which solutions can be developed. For example, applications consistently deficient in a specific area may signal the need for educational outreach and reviews that are consistently submitted late may provide impetus to recruit new Board members or reviewers.

### REB members

The primary responsibility for evaluating the substantive ethics issues in applications and how they are managed rests with the REB members and the Chair. The Board may approve applications, approve pending modifications, or reject them based on their compliance with standards and regulations.

Like administrators, an REB member’s efficiency and review quality are enhanced by the use of standard tools, in this case standardized review templates, intended to guide reviewers and Board members to address a consistent set of criteria. Where possible, matching members’ expertise to the application to be reviewed also contributes to timely, good quality reviews.

REB functioning is enhanced with ongoing member training and education, yielding consistent, efficient application of ethics principles and regulatory standards [15]. This may be undertaken in a variety of ways, including Board member retreats, regular circulation of current articles, and attending presentations and conferences. REB Chairs are accountable to ensure consistency in the decisions made by the Board (TCPS 2014, Article 6.8). This demands that Chairs thoroughly understand ethical principles and regulatory standards and that they maintain awareness of previous decisions. Much time can be spent at Board meetings covering old ground. The use of REB decision banks has been recommended as a means of systematizing a record of precedents, thus contributing to overall quality improvement [34].

### Institution

Where research ethics review takes place under the auspices of an academic institution, the institutions must typically take responsibility to adequately support the functioning of their Boards and promote a positive culture of research ethics [3, 5]. Supporting the financial and human resource costs of participating in ongoing education (e.g. retreats, speakers, workshops, conferences) is therefore the responsibility of the institution.

Operating an REB is costly [35]. It is reasonable to assume that there is a relationship between the adequacy of resources allocated to the workload and flow and the time to an REB decision. Studies have demonstrated wide variability in times to determination [8–10, 22]. However, comparisons are difficult to make because of confounding factors such as application volume, number of staff, number of REB members, application quality, application type (e.g. paper vs. electronic), and protocol complexity. Despite these variables, it appears that setting a modal target turnaround time of 6 weeks (±2 weeks) is reasonable and in line with the targets set in the European Union and the UK’s National Health Service [36, 37]. Tracking the time spent at each step in the model may reveal where applications are typically delayed for long periods and may be indicative of areas where more resources need to be allocated or workflows redesigned.

As institutions grow their volumes of research, workloads correspondingly increase for institutional REBs. To maintain service levels, institutions need to ensure that resources allocated to REBs match the volume and intensity of work. Benchmarking costs (primarily human resources) relative to the number of applications and time to a decision will help to inform the allocation of resources needed to maintain desired service levels.

Finally, most REB members typically volunteer their Board services to the institution. Despite their good-faith intent to serve, Board members occasionally find that researchers view them as obstacles to or adversaries in the research enterprise. Board members may believe that researchers do not value the time and effort they contribute to review, while researchers may believe the REB and its members are unreasonable, obstructive, and a “thorn in their side” [15]. Clearly, relationships can be improved. Nevertheless, improving the timeliness and efficiency of research ethics review should help to soothe fevered brows on both sides of the issue.

Upshur [12] has previously noted that the contributions to research ethics such as Board membership and application review need to be accorded the same academic prestige as serving on peer review grant panels and editorial boards and undertaking manuscript reviews. In doing so, institutions will help to facilitate a culture of respect for, and shared commitment to, research ethics review, which may only benefit the process.

## Conclusion

The activities, roles, and responsibilities identified in the ethics review model illustrate that it is a complex activity and that “the REB” is not a single entity. Multiple stakeholders each bear a portion of the accountability for how smoothly a research ethics application moves through the process. Time is used most efficiently when forward momentum is maintained and the application advances. Delays occur when the artefact (i.e. either the application or the application review) is not advanced as the accountable stakeholders fail to discharge their responsibilities or when the artefact fails to meet a standard and it is sent back. Ensuring that all stakeholders understand and are able to operationalize their responsibilities is essential. Success depends in part on the institutional context, where standards and expectations should be well communicated, and resources like education and administrative support provided, so that capacity to execute responsibilities is assured.

Applying this model will assist in identifying activities, accountabilities, and baseline performance levels. This information will contribute to improving local practice when deficiencies are identified and solutions implemented, such as training opportunities or reduction in duplicate activities. It will also facilitate monitoring as operational improvements over baseline performance could be measured. Where activities and benchmarks are well defined and consistent, comparisons both within and across REBs can be made.

Finally, this paper focused primarily on administrative efficiency in the context of research ethics review time. However, the identified problems and their suggested solutions would contribute not only to enhanced timeliness of review but also to enhanced quality of review and therefore human participant protection.

## References

[1] Beecher HK (1966). Ethics and clinical research. NEMJ.

[2] Kim WO (2012). Institutional review board (IRB) and ethical issues in clinical research. Korean J Anesthesiol.

[3] Canadian Institutes of Health Research, Natural Sciences and Engineering Council of Canada, and Social Sciences and Humanities Research Council of Canada. Tri-Council Policy Statement: Ethical Conduct for Research Involving Humans, December 2014. http://www.ethics.gc.ca/eng/index/. Accessed 21 Jun 2017.

[4] World Medical Association. Declaration of Helsinki: ethical principles for medical research involving human subjects as amended by the 64th WMA General Assembly, Fortaleza, Brazil, October 2013 U.S. Department of Health. https://www.wma.net/policies-post/wma-declaration-of-helsinki-ethical-principles-for-medical-researchinvolving-human-subjects/. Accessed 21 Jun 2017.

[5] U.S. Department of Health and Human Services, HHS.gov, Office for Human Research Protections. 45 CFR 46. Code of Federal Regulations. Title 45. Public Welfare. Department of Health and Human Services. Part 46. Protection of Human Subjects. Revised January 15, 2009. Effective July 14, 2009. Subpart A. Basic HHS Policy for Protection of Human Research Subjects. 2009. https://www.hhs.gov/ohrp/regulations-and-policy/regulations/45-cfr-46/. Accessed 21 Jun 2017.

[6] Abbott L, Grady C (2011). A systematic review of the empirical literature evaluating IRBs: what we know and what we still need to learn. J Empir Res Hum Res Ethics..

[7] Egan-Lee E, Freitag S, Leblanc V, Baker L, Reeves S (2011). Twelve tips for ethical approval for research in health professions education. Med Teach.

[8] Hicks SC, James RE, Wong N, Tebbutt NC, Wilson K (2009). A case study evaluation of ethics review systems for multicentre clinical trials. Med. J. Aust..

[9] Larson E, Bratts T, Zwanziger J, Stone P (2004). A survey of IRB process in 68 U.S. hospitals. J. Nurs. Scholarsh..

[10] Silberman G, Kahn KL (2011). Burdens on research imposed by institutional review boards: the state of the evidence and its implications for regulatory reform. Milbank Q..

[11] Whitney SN, Schneider CE (2011). Viewpoint: a method to estimate the cost in lives of ethics board review of biomedical research. J. Intern. Med..

[12] Upshur REG (2011). Ask not what your REB can do for you; ask what you can do for your REB. Can. Fam. Physician.

[13] Taylor H. Moving beyond compliance: measuring ethical quality to enhance the oversight of human subjects research. IRB. 2007;29(5):9–14. 17969778

[14] De Vries RG, Forsberg CP (2002). What do IRBs look like? What kind of support do they receive?. Account Res.

[15] Guillemin M, Gillam L, Rosenthal D, Bolitho A (2012). Human research ethics committees: examining their roles and practices. J Empir Res Hum Res Ethics.

[16] Grady C (2015). Institutional review boards: purpose and challenges. Chest.

[17] Burman WJ, Reves RR, Cohn DL, Schooley RT (2001). Breaking the camel’s back: multicenter clinical trials and local institutional review boards. Ann. Intern. Med..

[18] Whittaker E (2005). Adjudicating entitlements: the emerging discourses of research ethics boards. Health: An Interdisciplinary Journal for the Social Study of Health, Illness and Medicine.

[19] Turner L (2004). Ethics board review of biomedical research: improving the process. Drug Discov. Today.

[20] Nicholls SG, Hayes TP, Brehaut JC, McDonald M, Weijer C, Saginur R, et al. A Scoping Review of Empirical Research Relating to Quality and Effectiveness of Research Ethics Review. PLoS ONE. 2015;10(7):e0133639. doi:10.1371/journal.pone.0133639. 10.1371/journal.pone.0133639 PMC4520456 26225553

[21] Mansbach J, Acholonu U, Clark S, Camargo CA (2007). Variation in institutional review board responses to a standard, observational, pediatric research protocol. Acad. Emerg. Med..

[22] Christie DRH, Gabriel GS, Dear K (2007). Adverse effects of a multicentre system for ethics approval on the progress of a prospective multicentre trial of cancer treatment: how many patients die waiting?. Internal Med J.

[23] Greene SM, Geiger AM (2006). A review finds that multicenter studies face substantial challenges but strategies exist to achieve institutional review board approval. J. Clin. Epidemiol..

[24] Jester PM, Tilden SJ, Li Y, Whitley RJ, Sullender WM (2006). Regulatory challenges: lessons from recent West Nile virus trials in the United States. Contemp Clin Trials.

[25] Flynn KE, Hahn CL, Kramer JM, Check DK, Dombeck CB, Bang S (2013). Using central IRBs for multicenter clinical trials in the United States. Plos One.

[26] National Institutes of Health (NIH). Final NIH policy on the use of a single institutional review board for multi-site research. 2017. NOT-OD-16-094. https://grants.nih.gov/grants/guide/notice-files/NOT-OD-16-094.html. Accessed 21 Jun 2017.

[27] Check DK, Weinfurt KP, Dombeck CB, Kramer JM, Flynn KE (2013). Use of central institutional review boards for multicenter clinical trials in the United States: a review of the literature. Clin Trials.

[28] Dove ES, Townend D, Meslin EM, Bobrow M, Littler K, Nicol D (2016). Ethics review for international data-intensive research. Science.

[29] Ishikawa K. Introduction to Quality Control. J. H. Loftus (trans.). Tokyo: 3A Corporation; 1990.

[30] Nyeboer N (2014). Early-stage requirements engineering to aid the development of a business process improvement strategy.

[31] University of Calgary. Researchers. Ethics and Compliance. CHREB. 2017. http://www.ucalgary.ca/research/researchers/ethics-compliance/chreb. Accessed 21 Jun 2017.

[32] Harvard University. Committee on the Use of Human Subjects. University-area Institutional Review Board at Harvard. 2017. http://cuhs.harvard.edu. Accessed 21 Jun 2017.

[33] Oxford University. UAS Home. Central University Research Ethics Committee (CUREC). 2016. https://www.admin.ox.ac.uk/curec/. Accessed 21 Jun 2017.

[34] Bean S, Henry B, Kinsey JM, McMurray K, Parry C, Tassopoulos T (2010). Enhancing research ethics decision-making: an REB decision bank. IRB.

[35] Sugarman J, Getz K, Speckman JL, Byrne MM, Gerson J, Emanuel EJ (2005). The cost of institutional review boards in academic medical centers. NEMJ.

[36] National Health Service, Health Research Authority. Resources, Research legislation and governance, Standard Operating Procedures. 2017. http://www.hra.nhs.uk/resources/research-legislation-and-governance/standard-operating-procedures/. Accessed 21 June 2017.

[37] European Commission. Clinical Trials Directive 2001/20/EC of the European Parliament and of the Council of 4 April 2001. https://ec.europa.eu/health/sites/health/files/files/eudralex/vol-1/dir_2001_20/dir_2001_20_en.pdf. Accessed 21 Jun 2017.

